# Vortex-induced vibration wind energy harvesting by piezoelectric MEMS device in formation

**DOI:** 10.1038/s41598-019-56786-0

**Published:** 2019-12-31

**Authors:** Yin Jen Lee, Yi Qi, Guangya Zhou, Kim Boon Lua

**Affiliations:** 1 Department of Mechanical Engineering, National University of Singapore, Singapore, 117575 Republic of Singapore; 2 Department of Mechanical Engineering, National Chiao Tung University, Hsinchu, 30010 Taiwan, ROC

**Keywords:** Energy science and technology, Engineering, Nanoscience and technology

## Abstract

A silicon chip integrated microelectromechanical (MEMS) wind energy harvester, based on the vortex-induced vibration (VIV) concept, has been designed, fabricated, and tested as a proof-of-concept demonstration. The harvester comprises of a cylindrical oscillator attached to a piezoelectric MEMS device. Wind tunnel experiments are conducted to measure the power output of the energy harvester. Additionally, the energy harvester is placed within a formation of up to 25 cylinders to test whether the vortex interactions of multiple cylinders in formation can enhance the power output. Experiments show power output in the nanowatt range, and the energy harvester within a formation of cylinders yield noticeably higher power output compared to the energy harvester in isolation. A more detailed investigation conducted using computational fluid dynamics simulations indicates that vortices shed from upstream cylinders introduce large periodic transverse velocity component on the incoming flow encountered by the downstream cylinders, hence increasing VIV response. For the first time, the use of formation effect to enhance the wind energy harvesting at microscale has been demonstrated. This proof-of-concept demonstrates a potential means of powering small off-grid sensors in a cost-effective manner due to the easy integration of the energy harvester and sensor on the same silicon chip.

## Introduction

Recent interests in wireless sensor networks for Internet-of-Things applications have spurred researchers to study the methods of powering miniature off-grid devices, especially energy harvesting methods that can be easily integrated on silicon chips to provide long-term supply in a cost-effective manner. Here, we investigate the use of vortex-induced vibration (VIV) for miniature wind energy harvesting. In particular, we consider the use of multiple VIV wind energy harvesters in a dense formation to maximise the benefit of fluid-structure interaction to the power output of the device.

VIV is a fluid-structure interaction phenomenon that has been explored extensively in literature^1,2^. In brief, VIV occurs when periodic vortices, shed from a bluff body exposed to an incoming flow, induce periodic forcing and cause significant oscillations due to resonance. Hence, VIV is a means of converting flow energy of the surrounding fluid into the mechanical energy of the elastically mounted bluff body. The bluff body in this context is henceforth referred to as the “oscillator”. By including a device that converts the mechanical energy of the oscillator into electrical energy, VIV can be used for energy harvesting. Compared to conventional turbines, VIV mechanisms tend to have very simple structures. Additionally, the VIV approach integrates well with piezoelectric-based mechanical-to-electrical energy converters, which can be easily miniaturised compared to electromagnetic induced based alternators that tend to suffer high frictional losses at very small scales.

Comprehensive reviews^3,4^ have summarised recent attempts at miniaturising wind energy harvesters. However, very few miniature VIV wind energy harvesters of the dimensions of 10 mm × 10 mm × 10 mm or smaller were reported^5–7^. Here, we design, fabricate, and test a compact silicon-chip-integrated microelectromechanical (MEMS) VIV wind energy harvester that uses formation effect for effective energy harvesting. This study serves as a proof-of-concept of the above device, as well as a preliminary look into how the fluid-structure interaction and vortex interaction of multiple cylinders in formation serve to enhance VIV energy harvesting output.

On the subject of VIV devices in formation, the vortex shedding behaviour of two cylinders in cross-flow, arranged in in-line (also known as the ‘tandem’ in literature), side-by-side, and staggered formations is a classical fluid mechanics problem that has been much discussed in literature; see comprehensive reviews in^8–11^, among others. Of the three types of configuration, the in-line or tandem configuration is the most interesting for VIV energy harvesting applications, because the downstream cylinder is located in the wake of the upstream cylinder. The vortex interaction arising from such arrangement may be a means of increasing the fluctuating fluid forces acting on the downstream cylinder, resulting in an increase in VIV response and power output.

For two cylinders in in-line arrangement, past studies^8,10,11^ have shown that the flow structures are mainly dependent on the longitudinal pitch ratio, *L/D*, in which *L* is the centre-to-centre distance between upstream and downstream cylinders and *D* is the cylinder diameter. At very low *L/D* < 2, the extended body regime or single bluff body regime occurs, in which the two cylinders undergo vortex shedding as a single, elongated body. The reattachment regime occurs at the intermediate *L/D* range between a lower bound of around *L/D* = 2 and an upper bound of *L/D* = 4 to 5, depending on flow condition. In this reattachment regime, shear layers separating from the upstream cylinder reattaches onto the downstream cylinder. At sufficiently high *L/D* (above 4 to 5), the co-shedding regime occurs, in which vortex shedding occurs from both upstream and downstream cylinders. For the present application, the co-shedding regime appears most promising because the impingement of vortices from the upstream cylinder is likely to enhance the VIV response and power output of the downstream cylinder. Indeed, it has been shown in^12^ that, for two in-line cylinders in cross-flow, the fluctuating fluid forces acting on the downstream cylinder increases significantly when *L/D* > 4.

Hence, this study will focus on the VIV wind energy harvesting potential of cylinders arranged in in-line formations with *L/D* close to 4. This is a proof-of-concept study with the aim of demonstrating an effective method of enhancing the power output of microscale wind energy harvesters. Instead of using only two cylinders like most fundamental fluid mechanics studies in literature, our experiments use larger formations of up to 25 cylinders. A larger parameter space is explored using numerical simulations, in which formation sizes of up to 49 cylinders are considered. The large formation reflects our vision on how MEMS wind energy harvesters will be implemented in practice to power off-grid Internet-of-Things devices. For such applications, it is desirable to maximise power output within a very limited space, thus a large number of energy harvesters will be distributed over all usable surface area. As far as the authors are aware, the use of such formation effect as a means of enhancing the power output of wind energy harvesters have not been demonstrated in the past.

## Results

### MEMS energy harvester design

**Figure 1:**
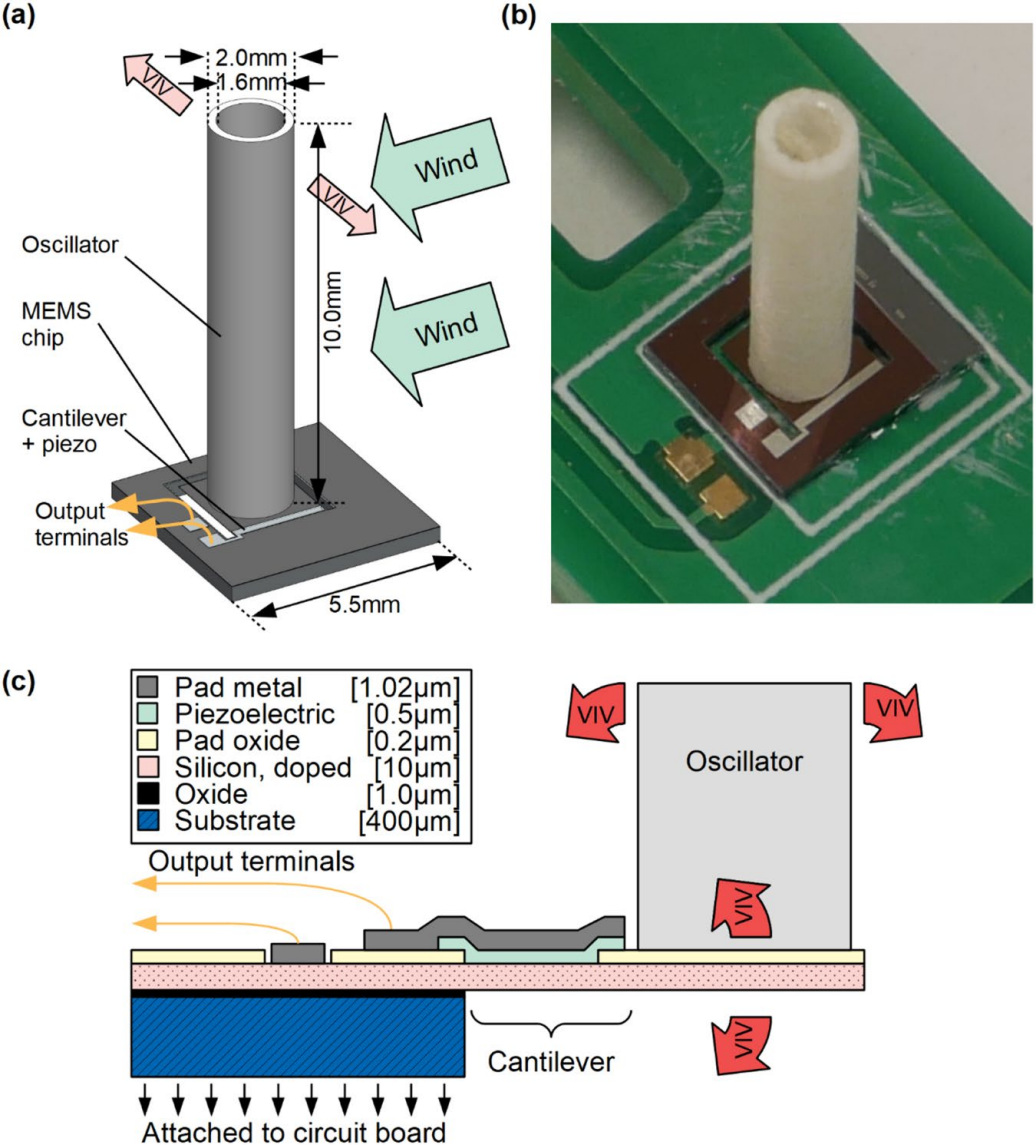
(**a**) Schematic drawing of the energy harvester, (**b**) photograph of the energy harvester, (**c**) schematic drawing of the MEMS device showing individual device layers.

The energy harvester comprises of a circular cylindrical oscillator (hollow polystyrene) attached by cyanoacrylate glue to a MEMS platform (Fig. 1(a,b)). The MEMS device is fabricate using MEMSCAP's PiezoMUMPs 5-mask level patterning and etching process. The device layers produced by this process is detailed in Fig. 1(c). The device comprises of the oscillator platform which is suspended by a silicon cantilever. A layer of piezoelectric material (aluminium nitride, AlN) on the cantilever serves as a means of converting mechanical energy into electrical energy. In the present design, the AlN layer operates in the *d*_31_ piezoelectric mode; although this piezoelectric material and mode has lower power output compared to some piezoelectric ceramics such as lead zirconate titanate (PZT), it does not require polarisation and has a relatively simple fabrication process^13^. Hence, AlN is a feasible choice for our intended application, namely, mass-producible silicon-chip-integrated energy harvester. Deformation of the piezoelectric layer generates alternating current, which is conducted by an aluminium/chrome layer to the output terminals; these are connected to a printed circuit board (PCB) via gold wire bonding.

**Figure 2:**
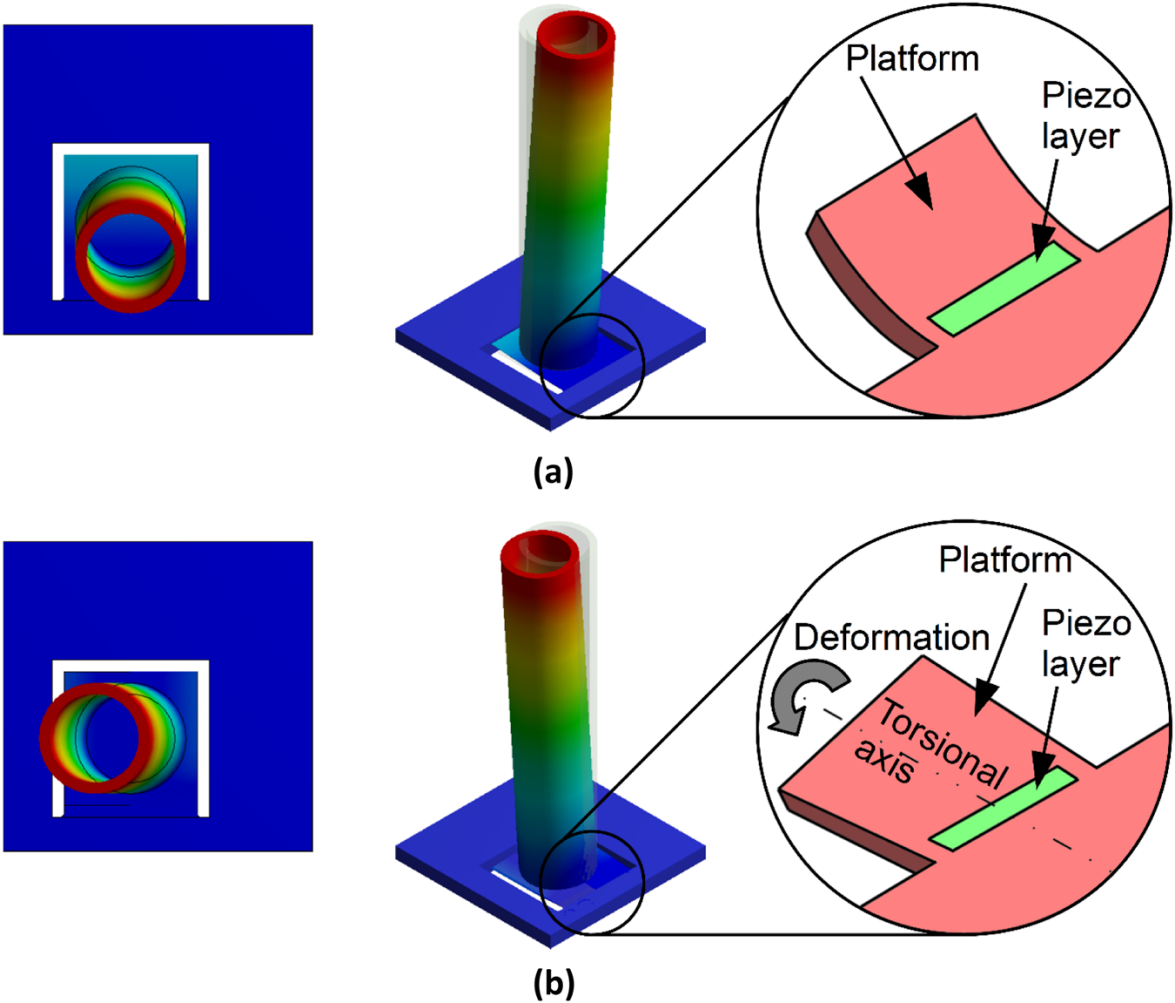
Modal analysis results showing the two vibration modes of the energy harvester. (**a**) Mode 1 deformation, (**b**) Mode 2 deformation.

Numerical modal analysis on the MEMS device indicates the presence of two vibration modes, which are shown in Fig. 2. Mode 1, shown in Fig. 2(a), occurs at 58 Hz and the piezoelectric cantilever undergoes bending deformation. Mode 2, shown in Fig. 2(b), occurs at 154 Hz and the piezoelectric cantilever undergoes torsional deformation. Of the two vibration modes, only Mode 1 is desirable; the bending of the piezoelectric layer creates a potential difference that causes in a flow of current when the output terminals of the MEMS device are connected to an external load, thus resulting in useful power output.

### Power output in isolation and in formation

**Figure 3:**
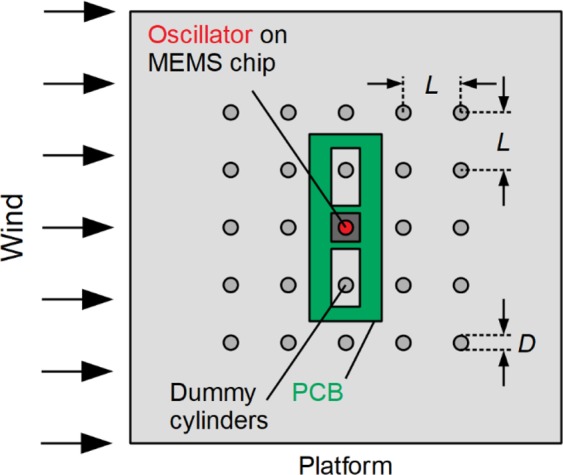
Schematic drawing of the energy harvester within a formation of stationary dummy cylinders.

The output terminals of the MEMS device are connected via the PCB to a 10 MΩ load resister, and the transient voltage across the load resistor is measured to compute the instantaneous power, *P*. *P* is obtained from wind tunnel experiments, in which the energy harvester is placed either in isolation or within a formation of stationary dummy cylinders made of solid polystyrene rods (see Fig. 3). Taking the time average of *P* over a sampling duration of one minute yields the average power output, *P*_*avg*_. The energy harvester in isolation is also referred to as the ‘1 × 1’ formation. For experiments in which the energy harvester is placed in formation, two different formation sizes have been tested, namely, the 3 × 3 and 5 × 5 formation. Two values of normalised cylinder-to-cylinder spacing, *L/D*, are considered, namely, *L/D* = 4.0 and 5.0 (see Fig. 3).

**Figure 4:**
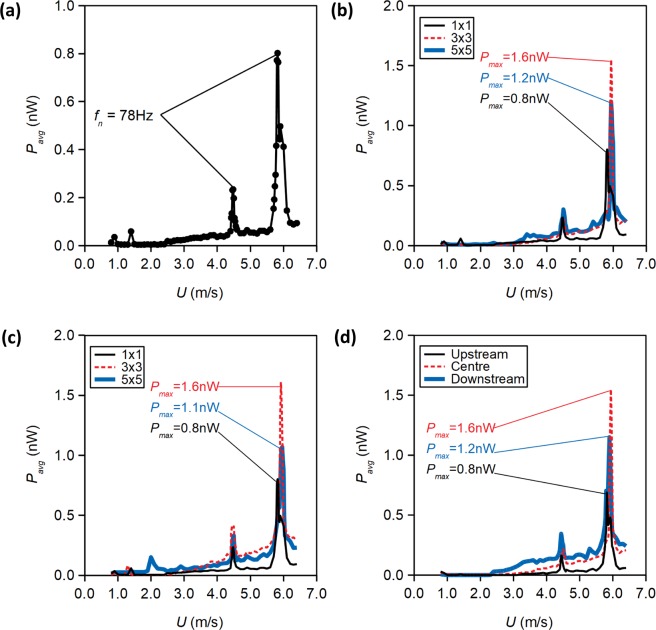
Power output of energy harvester at varying freestream velocity, under different formation effects. (**a**) 1 × 1 (isolated), (**b**) formation, *L/D* = 4.0, (**c**) formation, *L/D* = 5.0, (**d**) 3 × 3 formation, *L/D* = 4.0.

Figure 4(a) shows the *P*_*avg*_ obtained from a single energy harvester in isolation. Two distinct *P*_*avg*_ peaks are observed at wind tunnel speed *U* = 4.48 m/s and *U* = 5.82 m/s. At both *P*_*avg*_ peaks, Fourier analysis of the transient voltage values across the load resistor indicates that oscillation is occurring predominantly at *f*_*n*_ = 78 Hz. This *f*_*n*_ value is somewhat higher than the Mode 1 frequency of 58 Hz obtained from numerical modal analysis; this deviation can be primarily attributed to the uncertainty in material properties and exact dimensions of the MEMS chip after fabrication.

In the first series of wind tunnel experiments, the energy harvester is placed at the centre of the formation. For each formation, we measure *P*_*avg*_ over a range of wind tunnel speed, *U*, from 0.8 m/s to 6.4 m/s. The results, shown in Fig. 4(a–c), indicate that formation effect can significantly augment the trend of *P*_*avg*_ against *U*. The maximum value of *P*_*avg*_ obtainable within the velocity range considered, referred to as *P*_*max*_ in Fig. 4, is significantly affected by formation effects. Notably, we see that the energy harvester in formation yields considerable higher *P*_*max*_ than the same energy harvester in isolation; in particular, for the 3 × 3 formations, the formation effect can cause *P*_*max*_ to double. We also observe that the energy harvester in the centre of the 3 × 3 formation yields higher *P*_*max*_ compared to the same energy harvester in the centre of the 5 × 5 formation. Also, note that the *L/D* = 4.0 and 5.0 formations have almost identical effects on the power output.

Additionally, it is of interest to know whether the location of the energy harvester in a formation affects the power output. Hence, we conducted a second series of wind tunnel experiments, in which the arrangement of the dummy cylinders are changed so that the energy harvester becomes the upstream cylinder (i.e. first row) and the downstream cylinder (i.e. third row) in the 3 × 3 formation with *L/D* = 4.0. The results, shown in Fig. 4(d), indicate that, the energy harvester placed at the first row (most upstream) of the formation yields almost identical *P*_*max*_ compared to the energy harvester in isolation. *P*_*max*_ augmentation is most noticeable if the energy harvester is in the second row in the formation. Conversely, the energy harvester placed in the third row (most downstream) encounters a smaller *P*_*max*_ increase.

### Numerical analysis of harvester formation

The experimental measurements have successfully demonstrated the benefits of placing micro-scale VIV wind energy harvesters in a formation. However, the amount of information that we can gain from the experimental measurements is limited due to several technical challenges. Firstly, noticeable difference in behaviour is observed between individual devices in terms of resonance frequency and magnitude of power output. Thus, it is difficult to compare the power output between different devices; likewise, the velocity range at which peak power output is obtained differs between devices. Hence, in the analysis of our experimental data, we limit the comparison to looking at the power output of one individual device under different conditions, namely, different formations and incoming velocity. Furthermore, the MEMS device is fragile and is susceptible to damage due to mechanical shocks during the dismantling, transportation, and reinstallation processes which occur when we change the energy harvester formation between experiments. As a result, the parameter space that we can cover in the experiments is limited, and we are unable to evaluate the power output of every individual cylinder in the formation.

**Figure 5:**
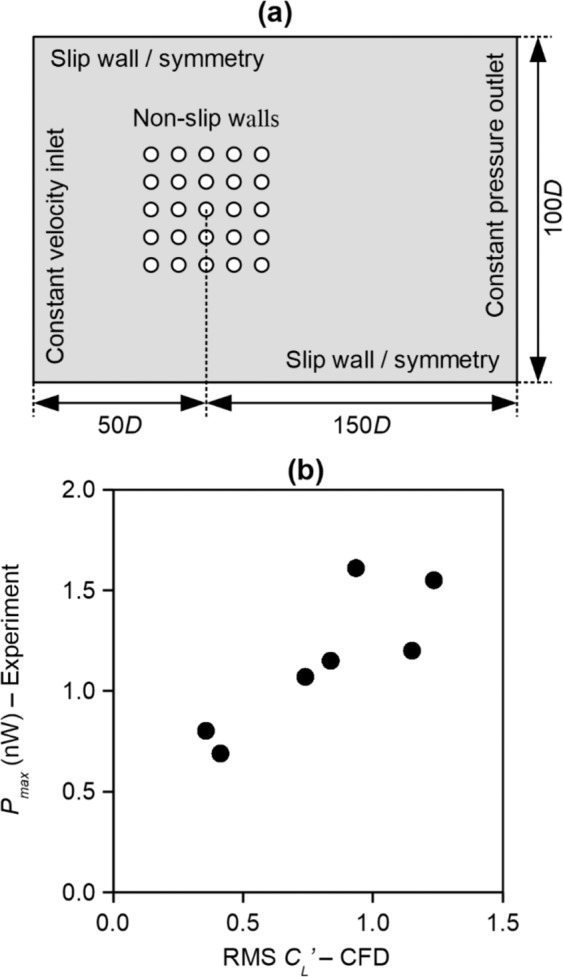
(**a**) CFD simulation setup and (**b**) comparison of *P*_*max*_ obtained from experiments and RMS *C*_*L*_’ obtained from CFD.

Hence, we conduct computational fluid dynamics (CFD) simulations to obtain more information. The purpose of CFD is threefold, namely, (i) to investigate whether the beneficial formation effect applies to all of the cylinders in formation, (ii) to explore cylinder formations and *L/D* values that have not been explored in wind tunnel experiments, and (iii) to gain insight on the fluid dynamics that result in the performance enhancement. Two-dimensional CFD simulations are conducted based on the simulation setup shown in Fig. 5(a). Note that the simulations are simplified compared to the experiments in that (i) all cylinders are assumed stationary and (ii) 2D simulations assume the absence of complex flow about the cylinder tip. It will be shown in the subsequent discussion that despite these simplifying assumptions, force coefficients obtained from CFD has a reasonable correlation with the power output measured from experiments, and therefore CFD results can be used to provide further insight.

The formations simulated in CFD include the 3 × 3, 5 × 5, and 7 × 7 formations with *L/D* = 3.0, 4.0, and 5.0. The range of *L/D* values are determined based on several known facts. According to literature^12^, the fluctuating forces acting on two cylinders in tandem undergo a significant increase when *L/D* is increased from *L/D* = 3.0. Thereafter, further increase in *L/D* decreases the fluctuating force because the interaction between cylinders decrease as *L/D* increases. Based on this information, we postulate that the fluctuating forces acting on cylinders in formation will be significantly enhanced within the range of 3 ≤ *L/D* ≤ 5, hence we limit our CFD studies to this range. From the simulations, the transient variation of the transverse force coefficient (*C*_*L*_) is obtained. *C*_*L*_ is the non-dimensional representation of the transverse force, and is normalised as shown in (1), in which *F*_*L*_ and *ρ* are the transverse force and fluid density, respectively. The root-mean-square of *C*_*L*_ fluctuation (RMS *C*_*L*_’) is taken as a measure of the power output potential of each cylinder in the formation, because the *F*_*L*_ is the key force component that gives rise to VIV. Indeed, Fig. 5(b) shows a strong correlation between *P*_*max*_ obtained from experiments and RMS *C*_*L*_’ obtained from CFD, thus justifying our use of RMS *C*_*L*_’ as a measure of the power output of each individual energy harvester in any given formation.

1 $${C}_{L}={F}_{L}/(0.5\rho D{U}^{2})$$

**Figure 6:**
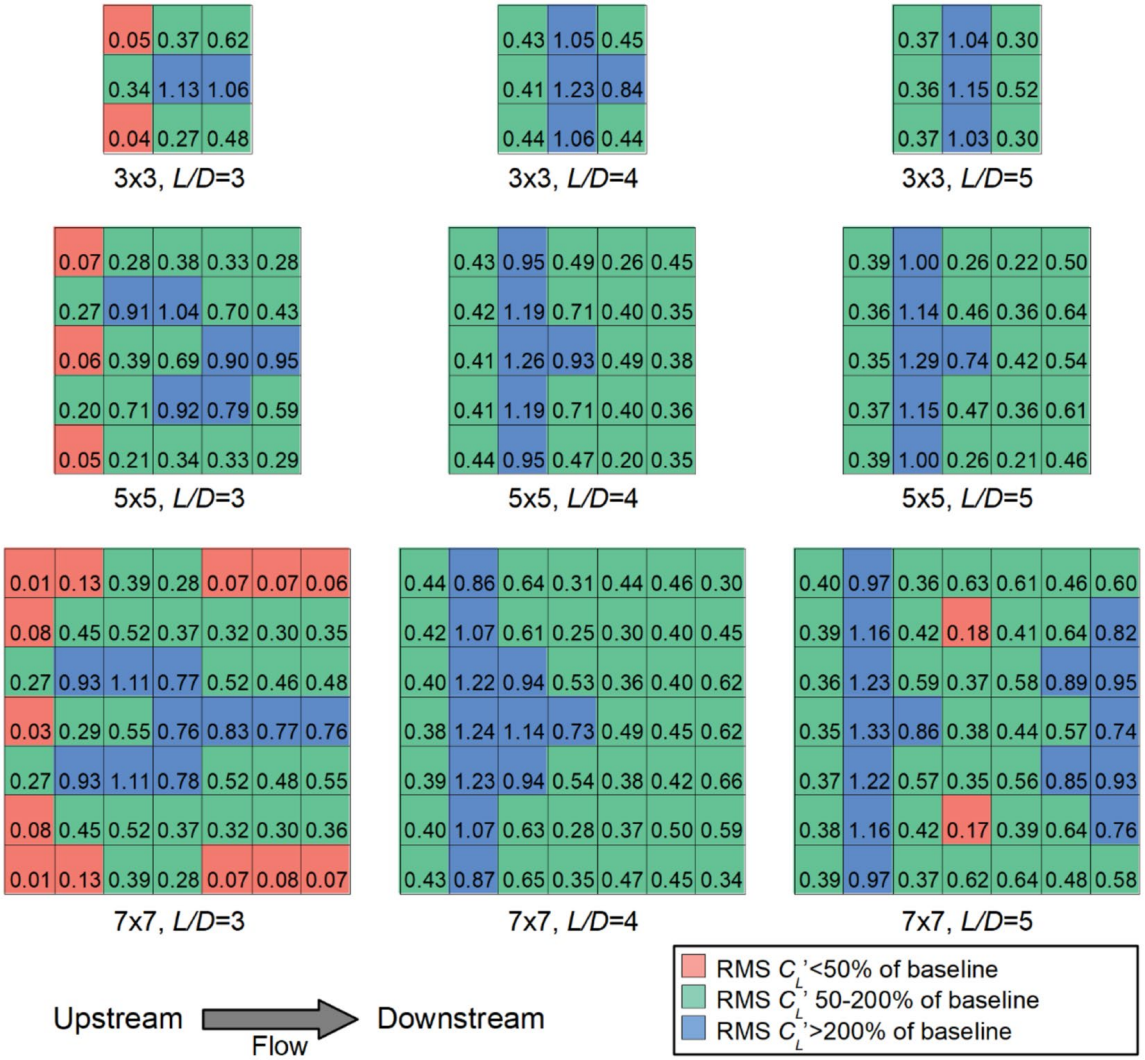
RMS *C*_*L*_’ for individual cylinders under different formation effects, obtained from CFD. Each grid represents one cylinder and the number within the grid indicates RMS *C*_*L*_’ for the cylinder in that particular position within the formation. Red grids indicate cylinders yielding significantly decreased RMS *C*_*L*_’ compared to the baseline (i.e. without formation effect); conversely blue grids indicate cylinders yielding significantly increased RMS *C*_*L*_’ compared to the baseline.

To get an indication on whether the power output of each individual cylinder in a given formation is enhanced by the formation effect, we plot the RMS *C*_*L*_’ value for every cylinder in the 3 × 3, 5 × 5 and 7 × 7 formations with *L/D* = 3.0, 4.0, and 5.0 (see Fig. 6). In Fig. 6, each grid represents one cylinder in the formation and shows the RMS *C*_*L*_’ value. The baseline of our comparison is the RMS *C*_*L*_’ of a single cylinder in isolation, which yields RMS *C*_*L*_’ = 0.36. Cylinders which encounter significantly decreased RMS *C*_*L*_’ (less than half of baseline) are denoted by the red grids and cylinders which encounter significantly increased RMS *C*_*L*_’ (more than double of baseline) are denoted by the blue grids in Fig. 6. Several observations can be made on how the formation affects the transverse force encountered by the VIV energy harvesters, and hence, their potential power output. In the following sections, we discuss the formation effects based on *L/D* ratio because this ratio appears to play a critical role in determining the formation effect.

### Flow field of *L/D* = 3.0 cylinder formation

For the *L/D* = 3.0 formations, the cylinders in the first or most upstream row typically encounter significantly lower RMS *C*_*L*_’ compared to baseline (see Fig. 6), with baseline RMS *C*_*L*_’ = 0.36. This is most obvious for the cylinders near the upstream corners of the formation. Conversely, some of the downstream cylinders can have significantly enhanced RMS *C*_*L*_’, reaching values more than 200% of baseline.

**Figure 7:**
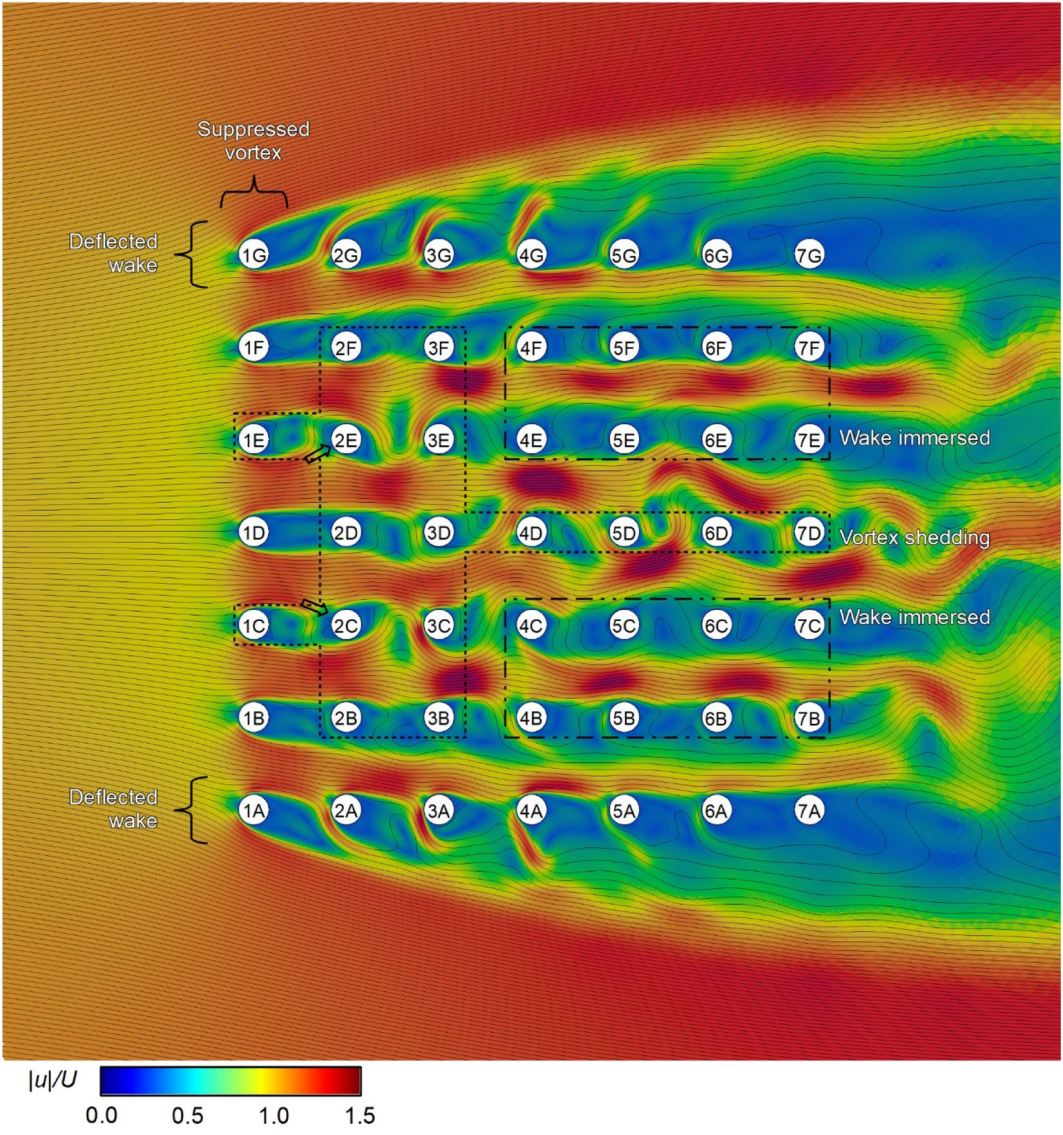
Contours of normalised velocity magnitude encountered by the cylinders in a 7 × 7 formation with *L/D* = 3.0. The fine black lines are streamlines which indicate the instantaneous flow direction at any point within the domain. Freestream flow is from left to right.

The reason behind this is best understood by looking at the flow field shown in Fig. 7, which shows the contours of normalised velocity for the 7 × 7 formation with *L/D* = 3.0. Here, the instantaneous local velocity magnitude, |*u*|, is normalised by the mean freestream velocity, *U*. The instantaneous streamlines, depicted by black lines in Fig. 7, show the flow direction. Looking at the cylinders in the first row (1 A to 1 G), it is apparent that most of the cylinders (1 A, 1 B, 1 D, 1 F, and 1 G) do not show the classical von Karman vortex shedding pattern that is typically encountered for a single isolated cylinder in cross-flow. We have labelled this behaviour as the “suppressed vortex” behaviour in Fig. 7. In particular, cylinders 1 B, 1 D, and 1 F show the “single bluff body regime” reported in past literature^8,10,11^, in which two in-line cylinders in close proximity undergo vortex shedding as a single, elongated body. Notably, the streamlines separating from the upstream cylinder reattaches at the downstream cylinder. As a result of this lack of vortex shedding, the RMS *C*_*L*_’ of cylinders 1 B, 1 D, and 1 F are close to zero (see Fig. 6). Cylinders 1 A and 1 G also do not show vortex shedding, and instead have elongated low velocity regions that are deflected outwards away from the centre of the formation (labelled as “deflected wake” behaviour in Fig. 7). Likewise, this lack of vortex shedding causes these corner cylinders to have very low RMS *C*_*L*_’. Conversely, cylinders 1 C and 1 E display vortex shedding behaviour (Fig. 7) and have RMS *C*_*L*_’ that are close to baseline (Fig. 6); we have labelled these cylinders as having the “vortex shedding” behaviour in Fig. 7.

Moving on to the second row (2 A to 2 G), we can see how the incoming flow caused by the upstream cylinders impact the RMS *C*_*L*_’ of the second row cylinders. Cylinders 2 C and 2 E are downstream of the vortices shed by cylinders 1 C and 1 E. As a result, cylinders 2 C and 2 E encounter incoming flow velocity that has a significant transverse component as shown by the streamlines (see also the hollow black arrows in Fig. 7). The periodic transverse velocity component of the flow induces a strong periodic force on the cylinders, resulting in high RMS *C*_*L*_’ values for cylinders 2 C and 2 E as seen in Fig. 6. Conversely, the other cylinders in row 2 which do not encounter vortices shed from the upstream cylinders (Fig. 7) do not have enhanced RMS *C*_*L*_’ compared to baseline (Fig. 6). The outer cylinders 2 A and 2 G encounter a steady incoming flow that is deflected outwards from the formation centre (Fig. 7) which result in very low RMS *C*_*L*_’ (Fig. 6). Note that unlike the incoming flow for cylinders 2 E and 2 C, which originate from shedding vortices and therefore have a periodically varying transverse velocity component, the incoming flow for cylinders 2 A and 2 G have a steady transverse velocity component that does not contribute to RMS *C*_*L*_’. It is notable that of the second row cylinders, cylinders 2 C and 2 E show very strong vortex shedding behaviour which is evident from the streamlines and the very obvious regions of minimum local velocity (Fig. 7). Conversely, the streamlines behind cylinders 2 B, 2 D, and 2 F show some characteristics of vortex shedding but these vortices do not appear to be as strong and obvious compared to those shed from cylinders 2 C and 2 E.

Subsequently, for row 3, we observe that cylinders 3 C and 3 E, which encounter very strong vortices shed from cylinders 2 C and 2 E (Fig. 7), have significantly enhanced RMS *C*_*L*_’ compared to baseline (Fig. 6). Cylinders 3 B, 3 D, and 3 F encounter weaker vortices shed by cylinders 2 B, 2 D, and 2 F (Fig. 7) and have RMS *C*_*L*_’ that are slightly higher than baseline (Fig. 6).

From row 4 onwards, RMS *C*_*L*_’ generally decreases as we move further downstream (Fig. 6). This is because the cylinders in row 4 and above tends to be immersed in the low velocity wakes created by the upstream cylinders; these appear as long continuous blue regions in Fig. 7. Because the transverse force scales with the square of incoming velocity, RMS *C*_*L*_’ tends to be lower for cylinders immersed in low velocity wakes. We labelled these cylinders as cylinders with “wake immersed” behaviour in Fig. 7. One exception is for the cylinders in column D (1 D to 7 D); it appears that cylinders in column D still have strong vortex shedding characteristics all the way to cylinder 7 D (Fig. 7), which cause enhanced RMS *C*_*L*_’ values for cylinders 4 D to 7 D (Fig. 6).

Overall, we see that formation effect is complex. Generally, for the *L/D* = 3.0 formation, most cylinders in the first row have the “suppressed vortex” behaviour due to the close proximity of the downstream cylinders and are ineffective at generating RMS *C*_*L*_’; hence they will be ineffective at generating power via the VIV. The side-by-side proximity of the cylinders obviously affect the flow behaviour as well because some of the cylinders in the first row do shed vortices. We can also observe that the cylinders at the outer sides of the formation tend to display the “deflected wake” behaviour and yield low RMS *C*_*L*_’. Most cylinders inside the formation in the second and third row have the “vortex shedding” behaviour and yield high RMS *C*_*L*_’; cylinders that are downstream of vortex shedding cylinders tend to have exceptionally high RMS *C*_*L*_’. From row 4 onwards, most cylinders tend to be immersed in a low velocity wake and RMS *C*_*L*_’ is decreased.

### Flow field of *L/D* = 5.0 cylinder formation

**Figure 8:**
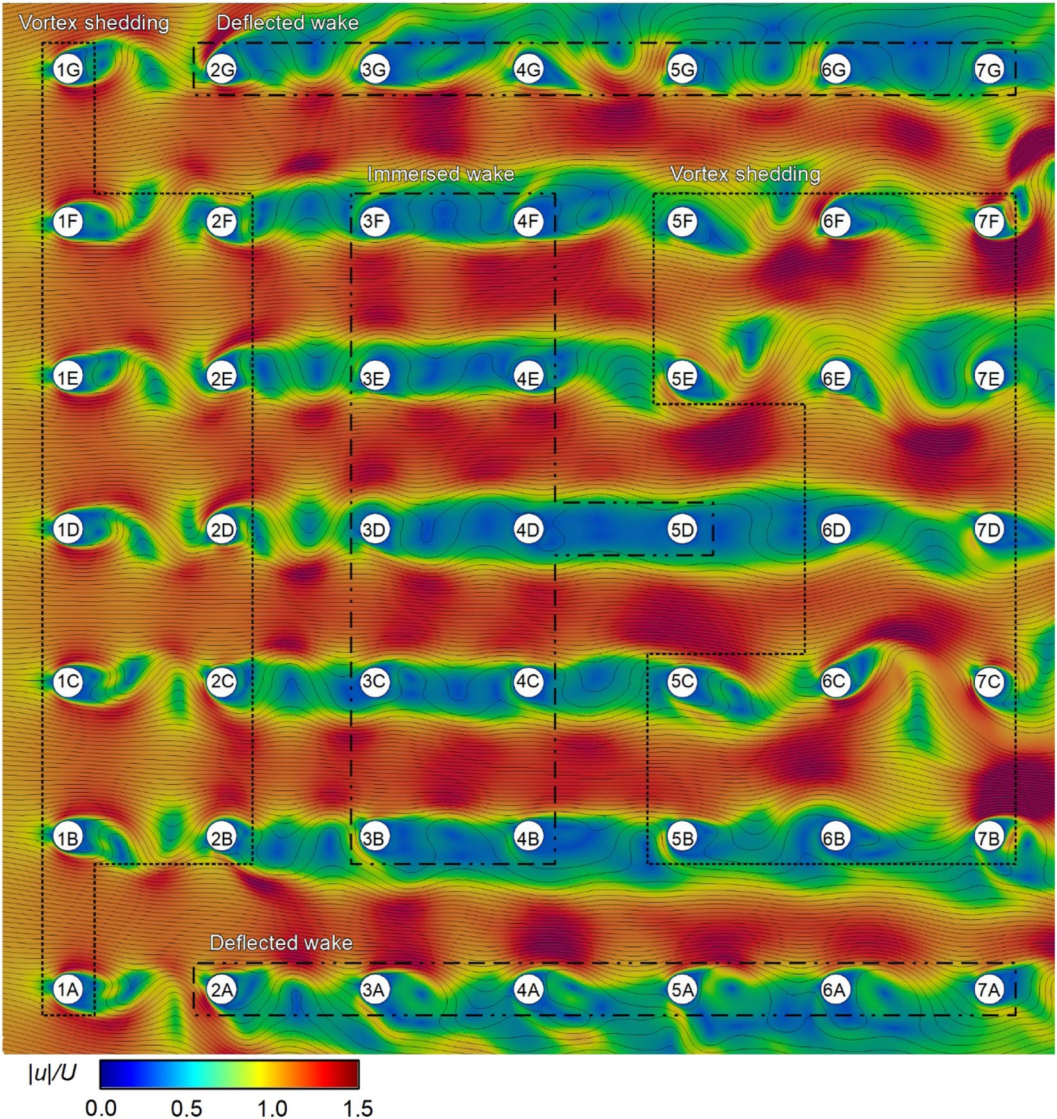
Contours of normalised velocity magnitude encountered by the cylinders in a 7 × 7 formation with *L/D* = 5.0. The fine black lines are streamlines which indicate the instantaneous flow direction at any point within the domain. Freestream flow is from left to right.

Because formations with *L/D* = 4.0 and 5.0 have rather similar characteristics, we shall group these formations together and draw general conclusions based on their collective behaviour. The flow field of the 7 × 7 cylinder formation with *L/D* = 5.0 is shown in Fig. 8.

We shall first study the first row of cylinders. Unlike the *L/D* = 3.0 formation (Fig. 7), we see that the cylinders 1 A to 1 G in the *L/D* = 5.0 formation display vortex shedding behaviour (Fig. 8). As a result, cylinders 1 A to 1 G have RMS *C*_*L*_’ comparable to baseline (Fig. 6). Moving onto the second row, as a result of the vortices shed from upstream cylinders (Fig. 8), cylinders 2 A to 2 G have significantly increased RMS *C*_*L*_’ compared to baseline (Fig. 6).

From row 3 onwards, the formation effect is more complex. The cylinders on the outer sides (2 G to 7 G and 2 A to 7 A) appears to show deflected wake (Fig. 8), which is similar to our observation for the *L/D* = 3.0 formation (Fig. 7). One major difference is that the deflected wake for *L/D* = 3.0 formation tends to have a steady flow devoid of vortices (Fig. 7); conversely, the deflected wake for *L/D* = 5.0 formation tends to be more complex with some vortices shed between cylinders (Fig. 8). This is likely attributed to the fact that at the higher *L/D* = 5, vortex formation is not suppressed by the proximity of the downstream cylinder, allowing complete vortices to be formed. Hence, for the present *L/D* = 5.0 formation, RMS *C*_*L*_’ of the cylinders on the outer sides tend to be comparable or slightly higher than baseline (Fig. 6). Within the formation, most of the row 3 and row 4 cylinders are immersed in the low velocity wake caused by the complex vortex interactions between the upstream cylinders (Fig. 8). As a result, RMS *C*_*L*_’ is not significantly higher than baseline; for cylinders 4 B and 4 F, RMS *C*_*L*_’ is in fact less than 50% of baseline (Fig. 6). Interestingly, further downstream, vortex shedding behaviour may be re-established; this can be observed for cylinders 5 B, 5 C, 6 D, 5 E, and 5 F. As a result, RMS *C*_*L*_’ for the two most downstream rows (rows 6 and 7) may again be significantly enhanced compared to baseline. Overall, we observe that at higher *L/D* = 5.0, formation effect is more beneficial because vortex suppression does not occur.

## Discussions

A silicon-chip-integrated VIV wind energy harvesting MEMS device has been tested. The MEMS device is connected to a load resistor and exposed to different wind speeds in a wind tunnel. Power output within the nanowatt range is measured, and noticeable increase in power output is observed when the energy harvester is placed within a formation of cylinders, compared to the energy harvester in isolation. Specifically, the maximum time-averaged power output can be doubled when the harvester is placed in a formation, compared to an identical harvester in isolation. The limitation of wind tunnel experiments is that, due to the limited number of MEMS devices available and their fragile nature, we are unable to explore a large parameter space, nor obtain the power output of every individual cylinder in each of the formations considered.

Further explorations are conducted using computational fluid dynamics (CFD) simulations. CFD allows exploration of more formations compared to the experiments. CFD also gives some indication on the effectiveness of each individual cylinder within the formation based on the magnitude of the fluctuation transverse force acting on each cylinder in the formation. By observing the flow field obtained from CFD simulations of cylinders in formation, the flow mechanism that gives rise to the enhancement effect is apparent. Vortices shed from upstream cylinders cause the downstream cylinders to encounter incoming flows with periodically varying transverse velocity components, which significant increases the transverse force encountered by the downstream cylinders, hence increasing the VIV response and power output. However, further downstream, cylinders may be immersed in the low velocity wake of the upstream cylinders, and the flow velocity encountered by the downstream cylinder decreases in magnitude. We also observed cases where, further downstream of the group of cylinders displaying wake immersed behaviour, the strong vortex shedding behaviour can be re-established near the end of the formation, causing the cylinders at the very downstream end of the formation to have unexpectedly high transverse force, hence increasing the VIV response and power output. Regardless of these counter-acting formation effects, it is consistent among all formations that the second row of cylinders are the most effective in terms of power output when used as VIV energy harvesters.

Overall, we observe that formation effect of the *L/D* = 3.0 formations are not as beneficial as the *L/D* = 4.0 or 5.0 formations, because the close proximity between cylinders in the *L/D* = 3.0 formation tends to suppress the formation of vortices. This is especially true for the first row of cylinders and the cylinders on the outer sides of the formation. Hence, in terms of how formation effect can benefit power output, *L/D* ≥ 4.0 is a better option.

It should be noted that the power output of this proof-of-concept device is not optimised and significant increase in the magnitude of power output is likely to be achieved if the piezoelectric layer of the MEMS device is sized appropriately to maximise power output. Our experiments have highlighted some technical challenges that need to be resolved in future work, including the fragile nature of the MEMS device, and the inconsistent behaviour between individual wind energy harvesters. For actual energy harvesting applications, more consideration needs to be given to the structural design and the manufacturing process control. Regardless of the challenges faced, this study demonstrates that the VIV wind energy harvester concept can be an attractive means of powering small off-grid sensors in a cost-effective manner due to the easy integration of the energy harvester and sensor on the same silicon chip; the power output can be significantly increased by making use of the formation effect.

## Methods

### Numerical modal analysis

Numerical modal analysis is conducted on a finite element model of the energy harvester using ANSYS structural analysis software, release 19.2. The MEMS device with oscillator is discretised into 9,937 elements (3D, 10-node tetrahedral solid elements with quadratic displacement behaviour). The silicon material that makes up the MEMS device is assumed to have a density, Young’s modulus, and Poisson’s ratio of 2330 kg/m^3^, 156 GPa, and 0.215, respectively. The polystyrene material that makes up the oscillator is assumed to have a density, Young’s modulus, and Poisson’s ratio of 1050 kg/m^3^, 45 MPa, and 0.34, respectively.

### Wind tunnel test

The power output of the MEMS VIV wind energy harvester is measured in a wind tunnel. The wind tunnel has a 4:1 contraction ratio and a 140 mm height by 590 mm width by 300 mm length test section. Turbulence intensity of the wind tunnel is measured using an in-house constant temperature anemometry hot-wire system with a frequency response of above 10,000 Hz. Turbulence intensity is below 0.3% throughout the range of velocity used in this study.

Experiments are conducted with the MEMS wind energy harvester mounted on a 50 mm tall platform, which ensures that the device is outside of the boundary layer created by the floor of the wind tunnel so that it encounters a uniform and steady stream of incoming velocity. The output terminals of the MEMS device are connected to a 10 MΩ load resistor, and the voltage across the load resistor is sampled at a frequency of 2,000 Hz using a data acquisition card. Power is computed based on *P* = *V*^2^*/R*.

MEMS device power output is measured at varying incoming velocity; the velocity is measured using a pitot tube connected to a pressure transducer with uncertainty of below 1% throughout the velocity range that is relevant to our study. The power output is measured at a sampling frequency of 2,000 Hz over the duration of 60s. Note that the all power output data presented in this study are obtained from a single MEMS device.

### Computational fluid dynamics (CFD) simulations

CFD simulations are conducted using the commercial solver ANSYS Fluent, release 16. The flow is assumed to be 2D, transient, and laminar. Note that the simulations are conducted in a non-dimensional framework that does not assume dimensional quantities for flow properties such as fluid density (*ρ*_*f*_), kinematic viscosity (*ν*), and flow velocity (*U*). Here, the flow properties are defined using the non-dimensional Reynolds number (*Re* = *ρ*_*f*_*U*/*ν*), which relates the inertial force and viscous force of the fluid. In our case, because we are conducting 2D flow simulations, we set the *Re* below the critical *Re* for the 2D-to-3D transition of flows over circular cylinders; it is well-established that for this type of flow, the flow transitions into inherently 3D flow when *Re* exceeds 190^14^. Hence, we set *Re* = 150, which is appropriate for our assumption of 2D flow.

The simulation setup is similar to^15^ and will not be repeated here. In brief, second order spatial and temporal discretisations are used for the fin ite volume discretisation. The computational mesh comprises of unstructured triangular ele ments with two layers of near wall rectangular elements. A simulation domain size of 100*D* (width) and 200*D* (length) is used. Validation results, available in^15^, indicate good agreement with past results in terms of force coefficients and Strouhal number for circular and non-circular cylinders in cross-flow.

In accordance to our non-dimensional simulation framework, the results obtained from the simulations are presented in the non-dimensional form; specifically, the fluid forces acting on the cylinder is presented using the non-dimensional transverse force coefficient (*C*_*L*_), and the flow field is analysed based on the contours of normalised velocity (*|u|/U*).

## Data Availability

The datasets generated during and/or analysed during the current study are available from the corresponding author on reasonable request.
